# Ticagrelor overcomes high platelet reactivity in patients with acute myocardial infarction or coronary artery in-stent restenosis: a randomized controlled trial

**DOI:** 10.1038/srep13789

**Published:** 2015-09-09

**Authors:** Pan Li, Yawei Yang, Tao Chen, Yu Liu, Ailin Cao, Junmei Liu, Zhuo Wang, Xianxian Zhao, Yongwen Qin, Liping Ma

**Affiliations:** 1 Department of Cardiology, Changhai Hospital, Second Military Medical University, 168 Changhai Rd, Shanghai, 200433, China; 2 Department of Pharmacy, Changhai Hospital, Second Military Medical University, 168 Changhai Rd, Shanghai, 200433, China

## Abstract

High on-treatment platelet reactivity (HTPR) is accompanied by an increased risk of adverse outcomes. Direct comparison of the antiplatelet effects between ticagrelor and high-dose clopidogrel has not yet been reported in acute myocardial infarction (AMI) or coronary artery in-stent restenosis (ISR) patients with HTPR. Consecutive patients with AMI or coronary artery ISR treated with standard-dose clopidogrel (75 mg/day) were screened with the VerifyNow assay, defining HTPR as P2Y12 reaction units (PRUs) >208. Of the 102 screened patients, 48 (47.06%) patients with HTPR were randomly assigned to either ticagrelor (180 mg/90 mg twice daily) or high-dose clopidogrel (150 mg/day) for 24 hours. Baseline characteristics and mean PRUs were similar in both groups. After 24 hours, ticagrelor was associated with a significantly lower platelet reactivity than high-dose clopidogrel (44.38 ± 40.26  vs. 212.58 ± 52.34 PRU, *P *< 0.05). No patient receiving ticagrelor exhibited HTPR, whereas 15 (62.50%) patients after treatment with high-dose clopidogrel remained HTPR (*P *< 0.05). During the follow-up (mean, 138.42 ± 53.59 days), no patient exhibited a major bleeding event in either treatment group. In conclusion, in patients with AMI or coronary artery ISR exhibiting HTPR after standard clopidogrel treatment, ticagrelor is significantly more effective compared with high-dose clopidogrel in overcoming HTPR.

Dual antiplatelet treatment with aspirin and clopidogrel is a cornerstone of therapy for patients with acute myocardial infarction (AMI) whether treated invasively or noninvasively^1^,^2^,^3^. Nevertheless, despite these antiplatelet agents, the frequency of recurrent thromboembolic events remains substantial (~10%)^4^. Recent data have reported inter-individual variability in response to clopidogrel^5^,^6^,^7^,^8^, and high on-treatment platelet reactivity (HTPR) has been associated with an increased risk of recurrent cardiovascular events in patients with AMI^9^,^10^. To overcome this problem, administration of a high daily dose of clopidogrel or adjunctive cilostazol to dual antiplatelet therapy is now occasionally used in clinical practice^11^,^12^. Despite this, an increased dose of clopidogrel or triple antiplatelet therapy did not reduce the incidence of cardiovascular death, accompanied with an increased risk of bleeding^13^,^14^,^15^.

Recently, new and more efficient alternative antiplatelet agents such as prasugrel and ticagrelor have been introduced into clinical application^16^,^17^. Unlike clopidogrel and prasugrel, ticagrelor is an orally active antagonist that reversibly binds to the P2Y12 platelet receptor, which yields faster, greater, and more consistent inhibition of platelet aggregation^18^. The Response to Ticagrelor in Clopidogrel Non-responders and Responders and Effect of Switching Therapies Study (RESPOND) demonstrated that ticagrelor therapy overcame the problem of HTPR during clopidogrel therapy in patients with stable coronary disease^19^. However, the prevalence of HTPR in Chinese patients with AMI or coronary artery in-stent restenosis (ISR) and the beneficial impact of ticagrelor compared with high-dose clopidogrel in overcoming HTPR are still unknown.

Here, we report the first prospective, randomised trial to be conducted in China to determine the antiplatelet effects of ticagrelor versus high-dose clopidogrel (150 mg) in AMI or coronary artery ISR patients with HTPR.

## Method

### Patient population and study design

We performed a prospective, randomised, single-center, single-blind study to compare the antiplatelet effects of ticagrelor versus high-dose clopidogrel (150 mg) in AMI or coronary artery ISR patients with HTPR. From April 2014 to November 2014, patients aged between 20 and 80 years hospitalised for ST Segment Elevation Myocardial Infarction (STEMI), non-(NSTEMI), or coronary artery ISR who were on aspirin and clopidogrel therapy were prospectively monitored for platelet reactivity (PR). Restenosis was defined as at least 50% in-stent diameter stenosis at the follow-up angiogram. Patients meeting all the inclusion criteria were consecutively enrolled into the study and treated and followed per the protocol in order to minimize selection bias. Exclusion criteria were (1) contraindication to antiplatelet therapy; (2) haemoglobin <10 g/dL or platelet count <100 000/mm^3^; (3) active bleeding and bleeding diatheses; (4) stroke within 6 months; (5) aspartate aminotransferase or alanine aminotransferase level >2 times the upper normal limit; (6) chronic renal failure (creatinine clearance <30 mL/min); and (7) active peptic ulceration or gastrointestinal bleeding. The study was performed in accordance with ethical principles that have their origin in the Declaration of Helsinki and are consistent with ICH/Good Clinical Practice. Informed consents were signed and obtained by all the patients. All experimental protocol was approved by the Ethics Committee of Changhai Hospital, Second Military Medical University. The trial was registered at Chinese Clinical Trial Registry. Trial registration No. was ChiCTR-RCS-14004303 and the date of registration was January 2, 2014.

**Figure 1:**
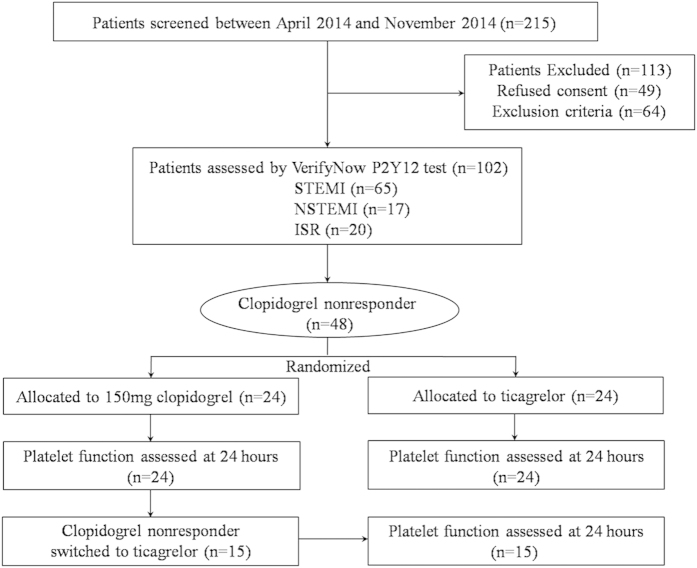
Patient flow chart.

A flow chart diagram of the study is shown in Fig. 1. Patients received a loading dose of 600-mg of clopidogrel for at least 6 hours, 300-mg of clopidogrel at least 12 hours, or were receiving chronic clopidogrel therapy (75 mg daily for ≥7 days) before the PR measurement. Patients received maintenance dose of 100 mg, once a day of aspirin and 75 mg, once a day of clopidogrel. In patients undergoing percutaneous coronary intervention (PCI), PR measurement was carried out after PCI. If patients received tirofiban or heparin therapy during PCI, tirofiban had to be discontinued for at least 48 hours, and heparin or low-molecular-weight heparin had to be discontinued for at least 12 hours before measurement of PR. Patients with HTPR (as defined subsequently) were randomized in a 1:1 ratio to receive either a high-dose clopidogrel of 150 mg daily or a loading-dose ticagrelor of 180 mg followed by 90 mg twice daily using a computer-generated randomisation table. A study nurse enrolled the participants and assigned participants to their groups. A second PR measurement was carried out 24 hours later in patients who received an alternate treatment. Patients who exhibited HTPR after being treated with high-dose clopidogrel were switched to 90 mg ticagrelor twice daily, and a third PR measurement was performed 24 hours after administration of ticagrelor.

### Clinical end points

Endpoints were pre-specified in the study protocol and statistical analysis plan. The primary end point of the study was PR after 24 hours of administration of ticagrelor versus high-dose clopidogrel, as determined by VerifyNow P2Y12 assay. The secondary end points included the rate of HTPR (defined as > 208 PRU). A clinical follow-up by telephone interviews was obtained at 1, 3, and 6 months and bleeding (major, minor, or minimal according to Thrombolysis in Myocardial Infarction [TIMI] criteria), and major adverse cardiovascular events (cardiovascular death, myocardial infarction, stent thrombosis, and stroke) were evaluated during the follow-up term.

### Blood sample collection

Blood samples were collected in 2 vacuum tubes (Becton-Dickinson, Franklin Lakes, NJ) containing 3.2% trisodium citrate, in which the first tube was discarded to avoid spontaneous platelet activation. The second tube was gently inverted 3 to 5 times to ensure complete mixing of the anticoagulant, and the blood sample was analysed within 4 hours for rapid platelet-function assay.

### Determination of HTPR

VerifyNow-P2Y12 (Accumetrics, San Diego, California) is a whole blood, rapid platelet function assay designed to measure the inhibition of clopidogrel on the P2Y12 receptor. Technical details of the platelet function assay were previously described^20^,^21^. Results were expressed as P2Y12 reaction units (PRUs), baseline value (BASE), and percent inhibition. The percent inhibition is calculated as follows: ([BASE-PRU]/BASE) × 100. A PRU of >208 was defined as HTPR or clopidogrel non-responder, linking the cut-off point to ischemic event occurrences^22^.

### Statistical analysis

For sample size calculation, we hypothesized that ticagrelor 90 mg, twice daily would result in a PR absolute difference of 50 PRU compared with clopidogrel 150 mg (with the assumption that the within-patient standard deviation of the response variable is 50 PRU), based on previously published data^19^,^23^. Choosing a power of 90% and a 2-sided alpha-level of 0.05, at least 46 patients in total (23 for each group) were required to reach statistical significance on the basis of the preceding assumptions. Continuous variables are reported as mean ± standard deviation (SD) and categorical variables are presented as frequencies and percentages. Continuous variables were compared by the unpaired Student *t*-test and categorical variables by the chi-square test or Fisher exact test. Analyses were performed using SPSS version 20.0 statistical software (SPSS Inc., Chicago, Illinois); *P*-value of < 0.05 were considered statistically significant.

## Results

### Baseline characteristics

Of the 102 (STEMI 63.73%, NSTEMI 16.67%, and ISR 19.61%) consecutive patients who were on the standard dose of clopidogrel therapy, 48 (47.06%) were identified to have HTPR, and randomly assigned to treatment with either high-dose clopidogrel (n = 24) or ticagrelor (n = 24). The demographics, clinical details, and concomitant medications were well balanced across the 2 treatment groups (Table 1).

### Laboratory characteristics

There were no significant differences between treatment groups with regard to laboratory characteristics (Table 2). After the standard dose of clopidogrel therapy, PR measured by the VerifyNow P2Y12 assay did not differ significantly in both groups (all *P *> 0.05).

### Angiographic characteristics

Of the 48 randomised patients, 47 (97.92%) patients underwent PCI and no patient received any revascularization. There were no significant differences in the angiographic characteristics between the 2 groups (Table 3).

### Platelet response to treatment modification

**Figure 2:**
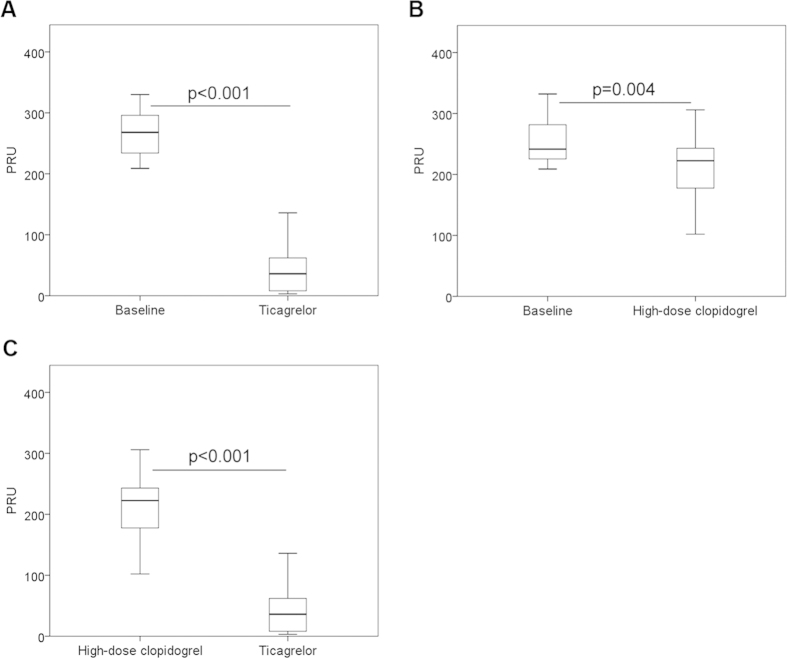
(**A**) Platelet reactivity (PR) in patients with high on-treatment platelet reactivity (HTPR) pre- and post-treatment with ticagrelor (180 mg/90 mg twice daily). (**B**) PR in patients with HTPR pre- and post-treatment with high-dose clopidogrel (150 mg/day). (**C**) PR in patients with HTPR post- treatment with high-dose clopidogrel (150 mg/day) or ticagrelor (180 mg/90 mg twice daily).

In the ticagrelor-treated group, the PRU level in patients with HTPR was significantly reduced from 269.04 ± 34.78 to 44.38 ± 40.26, 24 hours after administration of ticagrelor (*P *< 0.001; Fig. 2A), as determined by VerifyNow assay. In the high-dose clopidogrel group, the PRU values decreased from 252.71 ± 36.92 to 212.58 ± 52.34 after treatment with high-dose clopidogrel (*P *= 0.004; Fig. 2B). However, patients receiving ticagrelor achieved significantly lower PRU values compared with those who received high-dose clopidogrel (44.38 ± 40.26 vs. 212.58 ± 52.34, respectively; *P *< 0.001; Fig. 2C). Moreover, at the end of treatment periods, HTPR was still seen in 15 of 24 patients (62.50%) who received high-dose clopidogrel, but in no patients who received ticagrelor therapy (*P *< 0.001). In particular, for 15 patients with HTPR who were treated with high-dose clopidogrel, switching to ticagrelor led to further decrease in the PRU level (245.00 ± 28.57 vs. 30.91 ± 32.00, *P *< 0.05).

### Clinical events during the follow-up term

During the follow-up period of mean 138.42 ± 53.59 days, no patient exhibited major adverse cardiovascular events or a major bleeding event. 3 patients (under ticagrelor treatment) reported a mild new-onset dyspnoea, 1 patient (under ticagrelor treatment) reported diarrhoea, and 7 patients (4 under high-dose clopidogrel treatment and 3 under ticagrelor treatment) exhibited minimal bleeding.

## Discussion

To our knowledge, this is the first study in China to compare the effects of ticagrelor versus high-dose clopidogrel on platelet inhibition in AMI or coronary artery ISR patients with HTPR. The major findings of the study are (1) HTPR while on clopidogrel treatment was a common phenomenon found in about one half (47.06%) in our population; (2) the antiplatelet effect of ticagrelor was obviously greater than high-dose clopidogrel therapy in patients with HTPR; and (3) all patients treated with ticagrelor had PR below 208 PRU cut-off point, whereas more than half of patients (62.50%) receiving high-dose clopidogrel remained non-responsive to clopidogrel.

A number of studies have shown that the level of platelet inhibition achieved by clopidogrel varies considerably between individuals^24^. It has been estimated that nearly 4% to 30% of the patients exhibit low or no response to clopidogrel loading and maintenance therapy^6^,^8^,^25^,^26^. The antiplatelet effect of clopidogrel has been shown to be dose dependent, and has been examined to improve responses by increasing the dose^11^,^27^. However, persistent presence of HPPR was apparent despite high-dose of clopidogrel, and the clinical benefit is less clear. Compared with administration of clopidogrel 75 mg in patients with high-risk type 2 diabetes mellitus, clopidogrel 150 mg is associated with enhanced antiplatelet effects, but enhanced PR continues to persist in 60% of the patients on the 150-mg regimen^28^. In addition, the Gauging Responsiveness with A VerifyNow assay-Impact on Thrombosis And Safety (GRAVITAS) trial did not show any reduction in the incidence of death from cardiovascular causes, nonfatal MI, or ST with high-dose clopidogrel (600 mg LD, 150 mg/day MD) compared with standard-dose clopidogrel (no additional LD, 75 mg/day MD) in 2214 patients with HTPR after PCI with drug-eluting stents^29^.

Inter-individual variability in response to clopidogrel has prompted the development of novel P2Y12 inhibitors such as ticagrelor. Ticagrelor is the first oral P2Y12 receptor antagonist that blocks ADP-induced platelet aggregation in a reversible manner^18^. Unlike clopidogrel, it does not require activation via CYP450 enzymes because it is an active drug. In recent studies, ticagrelor has resulted in more potent inhibition of ADP-induced platelet aggregation than clopidogrel^18^,^30^. Bliden reported that compared with clopidogrel, ticagrelor was rapidly and consistently associated with a very low (0%–8%) prevalence of HTPR assessed with the VerifyNow assay^30^. In the PLATO trial, in patients with acute coronary syndrome (ACS), ticagrelor compared with clopidogrel significantly reduced the adverse event rate, without an increase in bleeding risk^31^. Notably, ticagrelor has been shown to be superior to high loading dose of clopidogrel in terms of reversing non-responsiveness to clopidogrel in patients with stable coronary artery disease^19^.

In the PLATO trial, the antiplatelet effects of ticagrelor in ACS patients being nonresponsive to clopidogrel are unknown. Also, the present study population differs from the RESPOND study that enrolled patients with AMI or coronary artery ISR; the clinical setting in which hyper-reactive platelets may be more considerable and the risk for ischemic complications is higher compared with stable coronary artery disease. Indeed, in the present study, 48 (47.06%) patients with HTPR were identified according to the VerifyNow system, a finding obviously significant than the previous reports in which the prevalence of clopidogrel to non-responsiveness was about 28%^8^,^19^. Other factors contributing to the high prevalence of HTPR in our population may include the definitions of PR cut-offs and the amount of clopidogrel loading dose. Nevertheless, whether the high rate of HTPR is related to the differences between races of human beings, large clinical trials might be needed to further proven.

High PR in patients with AMI has been shown predict the PCI angiographic success and recurrent atherothrombotic events^32^. Thus, the early and strong platelet inhibition seems to be of outmost importance, particularly in a population with high risk of ischemic events. Our study provides the first laboratory evidence that ticagrelor, compared with a high-dose clopidogrel of 150 mg/day may significantly eliminate HTPR in patients with AMI or coronary artery ISR. In the study, baseline rates of HTPR were 47.06%, which was reduced to 0 by switching from clopidogrel to ticagrelor compared with high-dose clopidogrel (31.25%). For the patients who were still resistant to high-dose clopidogrel, the level of PR was also decreased to below 208 PRU after switching to ticagrelor (*P *< 0.05). The results from this study suggest that in the presence of HTPR, switching to ticagrelor is a more reliably effective option for inhibiting platelet function compared with increasing the dose of clopidogrel. These findings have important clinical implications for guiding antiplatelet therapy in patients with high risk of cardiovascular events who are non-responsiveness to clopidogrel.

In recent years, an increasing number of studies have used VerifyNow P2Y12 assay to assess the antiplatelet effect of clopidogrel, and its relation to ischemic event occurrence during clopidogrel therapy has been demonstrated^33^. In our study, the change in PR was measured using VerifyNow P2Y12 assay as a point-of care test, with non-responder status predefined at >208 PRU and <85 PRU as thresholds to bleeding outcome^34^,^35^. In the present study, ticagrelor suppressed PR to a very low level (44.38 ± 40.26 PRU), which is far below the previous thresholds associated with bleeding risk, but without observing a major bleeding event. The low incidence of bleeding might be contributed to the sample size of the present study that is too small to assess the relation of PR with a bleeding event. However, the rate of bleeding in our study is in line with that was observed in the PLATO substudy, showing a relative low incidence of the bleeding event in Chinese patients^16^. Moreover, the data showed that overall bleeding events were similar in either treatment group, indicating that the benefits of ticagrelor were not associated with the increased rates of bleeding events compared with high-dose clopidogrel. Ticagrelor was more frequently accompanied by other mild side effects such as dyspnea and diarrhoea. This study had small sample size, therefore, large-scale randomised clinical trials and sufficient duration are required to confirm our findings, and to fully evaluate clinical outcomes, such as the incidence of stent thrombosis and major bleeding. Because of poor correlation between different tests of platelet function^36^, only single test of platelet function have been used in our study. However, the VerifyNow assay is most widely accepted test to assess the antiplatelet effects of P2Y12 receptor inhibitors, and has been shown to be useful in assessing the antiplatelet effects for identifying HTPR after clopidogrel or ticagrelor therapy^24^,^33^,^37^.

## Conclusions

In conclusion, the present results provide clinical evidence that, in patients with AMI or coronary artery ISR exhibiting HTPR after standard clopidogrel treatment, ticagrelor therapy is more effective than high-dose clopidogrel (150 mg) in reducing the PR and HTPR rate. The possible clinical benefits of this regimen require further validation.

## Additional Information

**How to cite this article**: Li, P. *et al.* Ticagrelor overcomes high platelet reactivity in patients with acute myocardial infarction or coronary artery in-stent restenosis: a randomized controlled trial. *Sci. Rep.* **5**, 13789; doi: 10.1038/srep13789 (2015).

## Figures and Tables

**Table 1:** Patient baseline characteristics.

	High-dose Clopidogrel Group (n = 24)	Ticagrelor Group (n = 24)	*P*-Value
Age, years	64.88 ± 9.75	68.08 ± 8.31	0.226
Male gender, n (%)	16 (66.67)	16 (66.67)	1.000
BMI, kg/m^2^	25.46 ± 3.53	23.51 ± 3.33	0.054
Abdominal circumference, cm	91.00 ± 9.14	88.38 ± 8.31	0.303
Diagnosis, n (%)			
MI	20 (83.33)	18 (75.00)	0.477
ST-elevation MI	11 (45.83)	16 (66.67)	
Non-ST-elevation MI	9 (37.50)	2 (8.33)	
ISR	4 (16.67)	6 (25.00)	0.477
Cardiovascular risk factors, n (%)			
Diabetes mellitus	5 (20.83)	8 (33.33)	0.330
Hypertension	16 (66.67)	15 (62.50)	0.763
Hyperlipidemia	1 (4.16)	4 (16.67)	0.161
Current smoking	14 (58.33)	10 (41.67)	0.248
History, n (%)			
Previous PCI or CABG	3 (16.67)	7 (25.93)	0.155
Previous MI	1 (4.16)	4 (16.67)	0.161
Previous stroke	0	2 (8.33)	0.153
Concomitant medications, n (%)			
Beta-blocker	9 (37.50)	9 (37.50)	1.000
ACEI or ARB	8 (33.33)	10 (41.67)	0.551
CCB	5 (20.83)	5 (20.83)	1.000
Statins	17 (70.83)	14 (58.33)	0.365
Tirofiban	8 (33.33)	12 (50.00)	0.242
GRACE score	139.46 ± 24.46	142.42 ± 19.88	0.648
CRUSADE score	29.17 ± 12.88	32.33 ± 12.58	0.393

BMI = body Mass Index, ACE I = angiotensin-converting enzyme inhibitor, ARB = angiotensin receptor blocker, CABG = coronary artery bypass graft, CCB = calcium channel blocker; ISR= in-stent restenosis, MI = myocardial infarction, PCI = percutaneous coronary intervention.

**Table 2:** Laboratory characteristics.

	High-dose Clopidogrel Group (n = 24)	Ticagrelor Group (n = 24)	*P*-Value
WBC (10^9^/L)	8.42 ± 3.26	9.72 ± 3.81	0.214
Hemoglobin (g/dL)	126.21 ± 14.91	126.38 ± 14.36	0.969
Platelet count (10^3^ μL)	206.92 ± 53.10	199.46 ± 59.10	0.648
Mean platelet volume (fl)	10.46 ± 1.19	10.13 ± 2.48	0.563
HbA1c, %	6.30 ± 1.26	6.45 ± 0.97	0.665
GFR (ml/min)	87.95 ± 25.21	88.15 ± 30.40	0.980
Total cholesterol (mg/dL)	4.12 ± 0.93	4.52 ± 1.07	0.182
LDL cholesterol (mg/dL)	2.29 ± 0.75	2.56 ± 0.92	0.283
Triglyceride (mg/dL)	1.31 ± 0.40	1.49 ± 0.70	0.293
PR before randomisation			
VERIFYNOW-P2Y12 PRU	252.71 ± 36.93	269.04 ± 34.78	0.122
VerifyNow BASE	302.04 ± 36.29	305.58 ± 47.51	0.773
VerifyNow% inhibition	16.38 ± 10.81	11.75 ± 13.01	0.187

GFR = glomerular filtration rate, LDL = low-density lipoprotein, PR = platelet reactivity, PRU = P2Y12 reaction unit; WBC = white blood cell.

**Table 3:** Angiographic characteristics.

	High-dose Clopidogrel Group (n = 24)	Ticagrelor Group (n = 24)	*P*-Value
Angiographic diagnosis			
No stenosis, n (%)	1 (4.17)	0	0.317
Single-vessel, n (%)	7 (29.17)	5 (20.83)	0.505
Multivessel, n (%)	16 (66.67)	18 (75.00)	0.525
Left main, n (%)	0	1 (4.17)	0.317
Severity of vascular lesions (%)			
<50%	2 (8.33)	0	0.153
50%–75%	1 (4.17)	0	0.317
75%–90%	10 (41.67)	7 (29.17)	0.365
>90%	11 (45.83)	17 (70.83)	0.079
Stent implantation (diameter, mm)	19 (79.17)	19 (79.17)	1.000
<30	15 (62.50)	10 (41.67)	0.149
>30	4 (16.67)	9 (37.50)	0.104
